# Pediatric demographics and regional trends from congenital anomalies of the kidney and urinary tract: A U.S. population-based study from 1999 to 2020

**DOI:** 10.1097/MD.0000000000047314

**Published:** 2026-01-23

**Authors:** Muhammad Ibrahim, Muhammad Husnain Ahmad, Hanzala Zahid, Malik Aqeel Ahmad, Fizza Inam, Fnu Sahil, Muhammad Khalid Afridi, Raheel Ahmad, Fatima Arshad, Muhammad Umair Ghafoor, Muhammad Bilal, Masab Ali

**Affiliations:** a Department of Internal Medicine, Faisalabad Medical University (Punjab Medical College), Faisalabad, Pakistan; b Department of Internal Mediicine, St Tentishev Asian Medical Institute, Kant, Kyrgyzstan; c Department of Internal Medicine, Liaquat University of Medical and Health Sciences, Jamshoro, Pakistan; d Department of Internal Medicine, Dow University of Health Sciences, Karachi, Pakistan; e Department of Internal Mediicine, Imperial College, London, United Kingdom; f Department of Internal Medicine, Quaid-e-Azam Medical College, Bahawalpur, Pakistan.

**Keywords:** congenital anomalies, epidemiology, health disparities, pediatric mortality, urinary tract malformations

## Abstract

Congenital malformations of the urinary system contribute significantly to pediatric morbidity and mortality. Understanding national and subgroup-specific mortality trends can guide targeted healthcare interventions and policy responses. Mortality data for individuals aged 0 to 14 years from 1999 to 2020 were obtained from the CDC WONDER database. Deaths attributed to congenital anomalies of the urinary system were identified using the International Classification of Diseases, Tenth Revision (ICD-10) codes Q60–Q64. Age-adjusted mortality rates (AAMRs) per 100,000 population were calculated, stratified by sex, race/ethnicity, geographic region, urban–rural classification, and state. Temporal trends in mortality were analyzed using Joinpoint regression models to estimate annual percentage changes and corresponding 95% confidence intervals. A total of 9412 deaths were recorded due to congenital anomalies of urinary system during the study period. The overall AAMR increased modestly from 0.61 in 1999 to 0.68 in 2020, representing an average annual percent change of + 0.62% (95% CI: −1.34 to 2.62) over the study period, with a significant decrease from 2007 to 2020 (APC: −1.36%; 95% CI: −2.01 to − 0.71). Males exhibited higher AAMRs than females (0.87 vs 0.49). Non-Hispanic Black (0.76) and Hispanic (0.70) children had AAMRs comparable to or higher than non-Hispanic Whites (0.70). The South region had the highest regional AAMR (0.79), and noncore rural areas showed the highest urbanization-specific AAMR (0.98). The District of Columbia recorded the highest state-level AAMR (1.35), while Massachusetts had the lowest (0.33). Despite an overall low and declining mortality rate, significant disparities persist across gender, race, geography, and urbanization. These findings highlight the need for equity-focused maternal-child health strategies to reduce preventable deaths from congenital urinary system anomalies.

## 1. Introduction

Congenital anomalies of the kidney and urinary tract (CAKUT) are structural anomalies of the kidneys and urinary system arising from disturbances in normal fetal development of the urinary tract.^[1]^ CAKUT is a leading cause of chronic kidney disease and end-stage renal disease in children. Studies indicate that CAKUT affects approximately 1 in 33 live births roughly 110,000 infants annually in the United States, imposing a substantial burden on patients, families, and healthcare systems.^[2]^ In 2019, birth defects were the leading cause of infant mortality, accounting for 4005 deaths, and generated an estimated $22.2 billion in hospitalization costs among individuals younger than 65 years.^[3,4]^ Congenital malformations of the urinary tract ranging from obstructive uropathies to renal dysplasia occur in over 1% of live births and are a major contributor to pediatric surgical interventions and long-term renal morbidity.^[1]^ Early detection and timely intervention are critical to improving renal outcomes and reducing complications. Despite advances in prenatal imaging and neonatal care, the burden of CAKUT-related mortality remains significant in pediatric populations.

Previous analyses have described national prevalence and hospitalization trends for selected birth defects from 1999 through 2020, yet mortality trends specific to urinary system malformations across demographic subgroups remain poorly characterized.^[5]^

To fill this critical knowledge gap, we performed a comprehensive 22-year retrospective analysis using Centers for Disease Control and Prevention’s (CDC) wide-ranging online data for epidemiologic research (WONDER) database to investigate national and subgroup-specific mortality trends from 1999 to 2020 in children under 14 years of age due to congenital malformations of the urogenital system. By offering the most up-to-date insights into disease burden and uncovering key disparities, this study aims to inform targeted clinical interventions and guide effective public health policy development.

## 2. Methodology

### 2.1. Study setting and population

Mortality data pertaining to CAKUT among individuals aged 0 to 14 years in the United States from 1999 to 2020 were obtained from the CDC WONDER Underlying Cause of Death database.^[6]^ This publicly accessible dataset compiles death certificate information from the National Center for Health Statistics beginning in 1999. Cases were identified using International Statistical Classification of Diseases and Related Health Problems–10^th^ Revision (ICD-10) codes (Q60–Q64), which cover congenital malformations of the kidney and urinary tract. These codes align with standard CAKUT definitions.^[1]^ While Q61 includes polycystic kidney disease, these represent congenital cystic malformations with significant pediatric mortality burden. Although data for all pediatric age categories were initially examined, the analysis was limited to individuals aged 0 to 14 years due to minimal mortality counts in older pediatric subgroups. The study adhered to the STROBE (Strengthening the Reporting of Observational Studies in Epidemiology) reporting standards. As the analysis utilized publicly available, de-identified data, institutional review board (IRB) approval was not required.^[7]^

## 3. Data abstraction

The following variables were extracted from the CDC WONDER database: year of death, sex, race/ethnicity, U.S. Census region, and county-level urbanization classification. Race and ethnicity were categorized as non-Hispanic (NH) White, NH Black or African American, Hispanic or Latino, NH Asian or Pacific Islander, and NH American Indian or Alaska Native. Due to consistently low annual case numbers and unstable mortality estimates, the NH Asian and NH American Indian or Alaska Native groups were excluded from the final analysis. Urbanization status was assigned, which includes large central metro, large fringe metro, medium metro, small metro, micropolitan, and noncore counties. Geographic regions were classified into Northeast, Midwest, South, and West based on U.S. Census Bureau regional divisions.

## 4. Statistical analysis

Crude mortality rates per 100,000 population were determined by dividing the number of deaths by the corresponding annual population estimates for each demographic category. Age-adjusted mortality rates (AAMRs) per 100,000 persons were calculated through direct standardization to the 2000 U.S. standard population,^[8]^ allowing for consistent comparisons across time periods and demographic subgroups. Temporal trends in mortality were evaluated using Joinpoint regression analysis (Joinpoint Regression Program version 5.0.2; National Cancer Institute, Bethesda, MD), which estimated annual percentage change and average annual percentage change, along with 95% confidence intervals. This log-linear regression model identifies statistically significant trend changes over time through the Monte Carlo permutation test.^[8]^ A 2-sided *P*-value of ≤.05 was considered indicative of statistical significance.

## 5. Results

From 1999 through 2020, there were 9412 documented deaths attributed to congenital malformations of the urinary system among individuals aged under 14 years. Of these, 6090 deaths (64.70%) occurred in males, while 3322 deaths (35.30%) were reported in females. A detailed summary of mortality counts is provided (Table S1, Supplemental Digital Content, https://links.lww.com/MD/R192).

## 6. Annual trends

**Figure 1.**
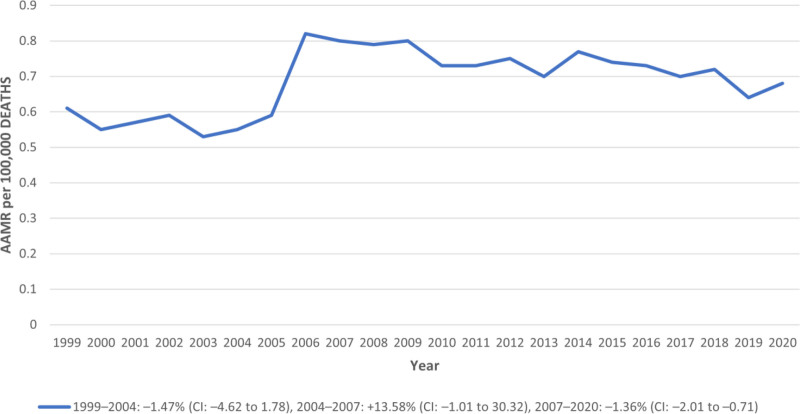
Annual trends in congenital malformation of urinary system AAMR. * indicates that the APC is significantly different from zero at the alpha = 0.05 level. AAMR = age-adjusted mortality rate, APC = annual percent change.

The AAMR remained relatively stable over the study period, with a slight significant increase from 0.61 per 100,000 (95% CI: 0.55–0.67) in 1999 to 0.68 (95% CI: 0.61–0.74) in 2020. The average annual percent change (APC) for the entire study period was + 0.62% (95% CI: −1.34 to 2.62; *P* = .536), indicating a nonsignificant trend. From 1999 to 2004, the AAMR showed a nonsignificant decline (APC:–1.47%; 95% CI: –4.62 to 1.78). This was followed by a transient increase from 2004 to 2007 (APC: +13.58%; 95% CI: –1.01 to 30.32), though the change did not reach statistical significance. A significant downward trend was observed between 2007 and 2020, with an APC of–1.36% (95% CI: –2.01 to–0.71). These findings indicate overall mortality remained low but exhibited modest fluctuations across the 2 decades (Table S2, Supplemental Digital Content, https://links.lww.com/MD/R192; Fig. 1).

## 7. Gender-specific trends

A statistically significant difference in AAMR between males and females was observed throughout the study period. Males consistently exhibited significantly higher AAMRs compared to females, with an overall rate of 0.87 per 100,000 (95% CI: 0.85–0.89) versus 0.49 (95% CI: 0.47–0.51) in females. In 1999, male AAMR stood at 0.75 (95% CI: 0.65–0.84) versus 0.48 (95% CI: 0.40–0.56) in females; by 2020, it was 0.88 (95% CI: 0.77–0.98) in males and 0.46 (95% CI: 0.38–0.54) in females.

For females, the trend was inconsistent: a nonsignificant decline from 1999–2003 (APC:–7.69%; 95% CI: –16.37 to 1.90), followed by a nonsignificant rise between 2003–2006 (APC: +18.90%; 95% CI: –16.74 to 69.80), and a marginally nonsignificant declining trend from 2006–2020 (APC:–1.21%; 95% CI: –2.52 to 0.12).

**Figure 2.**
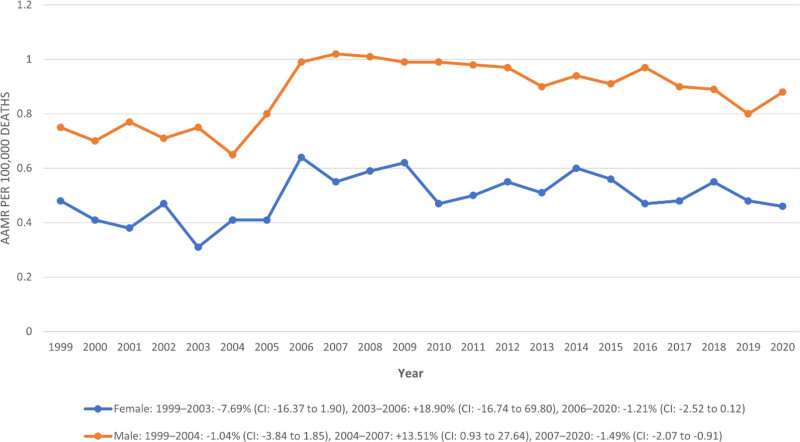
Trends in congenital malformation of urinary system AAMR stratified by sex. * indicates that the APC is significantly different from zero at the alpha = 0.05 level. AAMR = age-adjusted mortality rate, APC = annual percent change.

In contrast, males showed a sharper and more significant pattern: a mild nonsignificant decline from 1999–2004 (APC:–1.04%; 95% CI: –3.84 to 1.85), a significant spike between 2004–2007 (APC: +13.51%; 95% CI: 0.93–27.64), and a statistically significant downward trend from 2007–2020 (APC:–1.49%; 95% CI: –2.07 to–0.91) (Table S3, Supplemental Digital Content, https://links.lww.com/MD/R192; Fig. 2).

## 8. Race and ethnicity-specific trends

When analyzed by race and ethnicity, NH Black individuals exhibited the highest AAMR at 0.76 per 100,000 (95% CI: 0.72–0.80) and 18.61% of deaths. This was followed by the NH White population, with an AAMR of 0.70 (95% CI: 0.68–0.71), accounting for 76.95% of total deaths, and the Hispanic or Latino group, which had an AAMR of 0.70 (95% CI: 0.67–0.73) and comprised 25.30% of all deaths.

Among NH White individuals, mortality trends showed a nonsignificant decline from 1999 to 2004 (APC:–1.48%; 95% CI: –4.87 to 2.02), a nonsignificant rise from 2004 to 2007 (APC: +14.18%; 95% CI: –1.54 to 32.41), followed by a significant decline from 2007 to 2020 (APC: –1.48%; 95% CI: –2.17 to–0.79).

In the NH Black population, the trend was nonsignificantly stable from 1999 to 2003 (APC:–6.54%; 95% CI: –19.17 to 8.06), with a brief nonsignificant increase from 2003 to 2006 (APC: +24.79%; 95% CI: –16.03 to 85.45), followed by a statistically significant decline from 2006 to 2020 (APC:–1.71%; 95% CI: –3.29 to–0.10).

**Figure 3.**
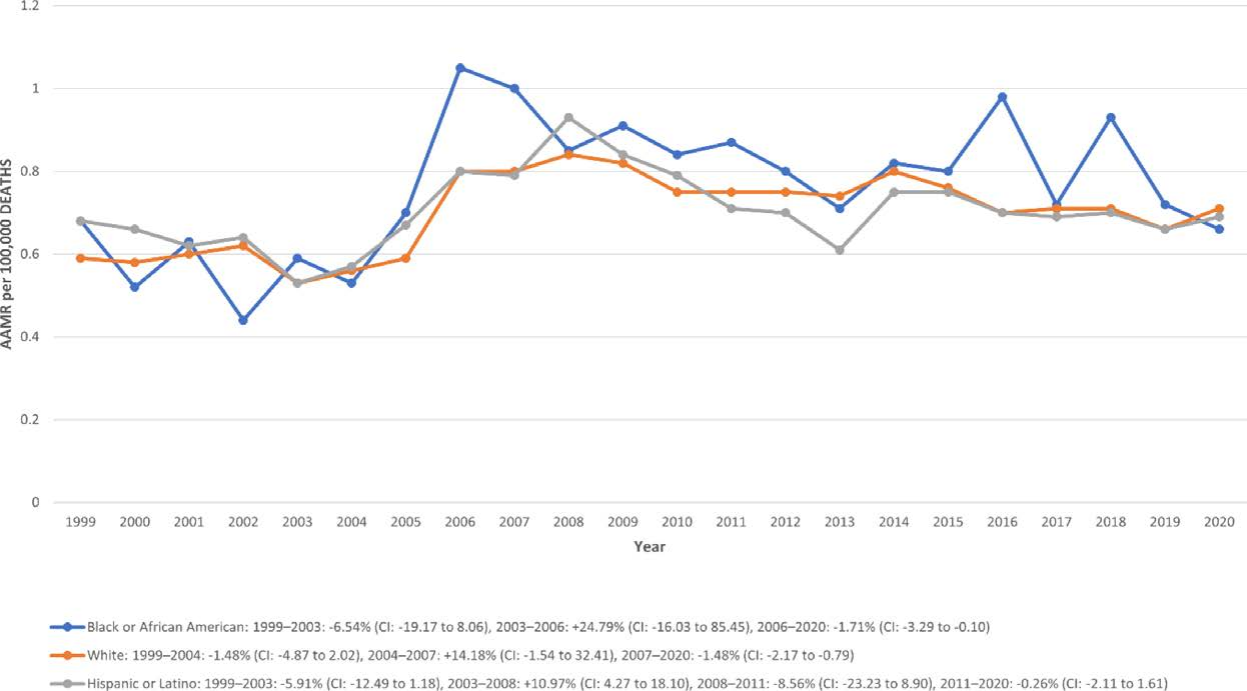
Trends in congenital malformation of urinary system AAMR stratified by race. * indicates that the APC is significantly different from zero at the alpha = 0.05 level. AAMR = age-adjusted mortality rate, APC = annual percent change.

For Hispanic or Latino individuals, mortality declined nonsignificantly from 1999 to 2003 (APC:–5.91%; 95% CI: –12.49 to 1.18), rose significantly from 2003 to 2008 (APC: +10.97%; 95% CI: 4.27–18.10), dropped again from 2008 to 2011 (APC:–8.56%; 95% CI: –23.23 to 8.90), and remained largely stable from 2011 to 2020 (APC:–0.26%; 95% CI: –2.11 to 1.61), though the changes was not statistically significant. (Table S4, Supplemental Digital Content, https://links.lww.com/MD/R192; Fig. 3).

## 9. State and region-based differences

**Figure 4.**
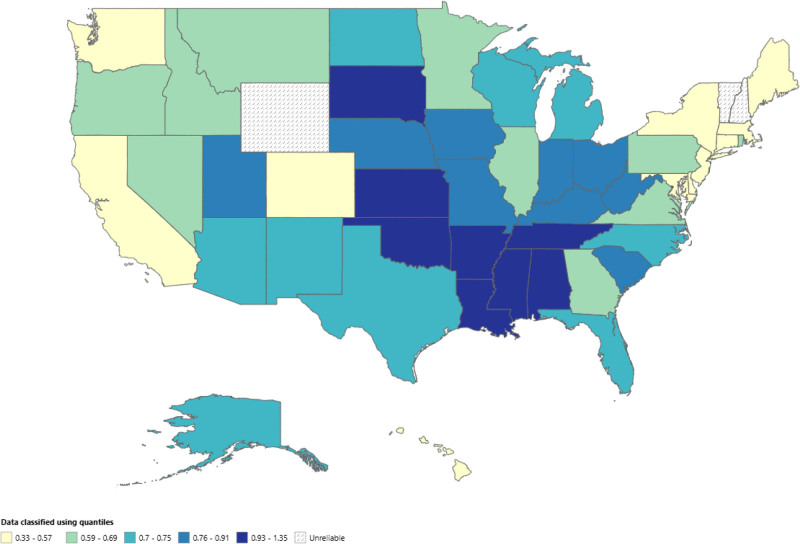
State-wise map highlighting the states with highest mortality rates in the United States from 1999 to 2020.

When stratified by state, substantial variation in AAMRs was observed across the United States. The highest AAMR was noted in the District of Columbia at 1.35 per 100,000 (95% CI: 0.95–1.85), followed by Mississippi (1.19; 95% CI: 1.01–1.37) and South Dakota (0.97; 95% CI: 0.68–1.33). In contrast, the lowest AAMRs were seen in Massachusetts (0.33; 95% CI: 0.26–0.41), New Jersey (0.41; 95% CI: 0.35–0.48), and Connecticut (0.44; 95% CI: 0.34–0.57). These findings indicate nearly a fourfold difference between the highest and lowest states. Regarding total burden, California reported the largest proportion of deaths (10.35%), followed by Texas (10.55%), Florida (5.81%), and New York (4.06%), whereas Vermont, Wyoming, and Delaware had sparse data with unstable estimates. (Table S6, Supplemental Digital Content, https://links.lww.com/MD/R192; Fig. 4)

**Figure 5.**
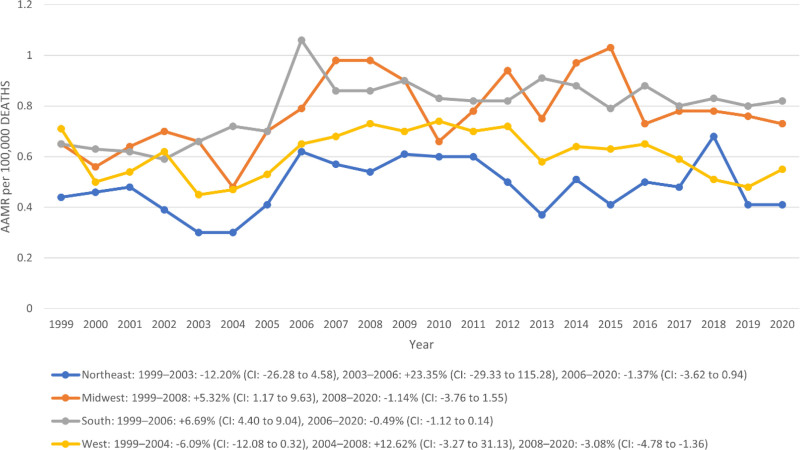
Trends in congenital malformation of urinary system AAMR stratified by region. * indicates that the APC is significantly different from zero at the alpha = 0.05 level. AAMR = age-adjusted mortality rate, APC = annual percent change.

Regional analysis by U.S. Census divisions showed that the South carried the highest mortality burden, with an AAMR of 0.79 per 100,000 (95% CI: 0.76–0.81), accounting for 43.42% of all deaths. The Midwest followed with an AAMR of 0.76 (95% CI: 0.73–0.79; 23.73%), then the West at 0.60 (95% CI: 0.57–0.62; 21.48%), while the Northeast reported the lowest AAMR at 0.48 (95% CI: 0.45–0.51), contributing 11.37% of total deaths. In the Northeast region, a nonsignificant decline occurred from 1999 to 2003 (APC:–12.20%; 95% CI: –26.28 to 4.58), followed by a short-term nonsignificant increase from 2003 to 2006 (APC: +23.35%; 95% CI: –29.33 to 115.28), and then a moderate nonsignificant decline from 2006 to 2020 (APC:–1.37%; 95% CI: –3.62 to 0.94). The Midwest experienced a significant rise from 1999 to 2008 (APC: +5.32%; 95% CI: 1.17–9.63), followed by a nonsignificant decline through 2020 (APC:–1.14%; 95% CI: –3.76 to 1.55). In the South, the significant rise occurred from 1999 to 2006 (APC: +6.69%; 95% CI: 4.40–9.04), and then plateaued with a slight nonsignificant decline between 2006 and 2020 (APC:–0.49%; 95% CI: –1.12 to 0.14). In the West, an initial nonsignificant decline was observed from 1999 to 2004 (APC:–6.09%; 95% CI: –12.08 to 0.32), followed by an nonsignificant increase between 2004 and 2008 (APC: +12.62%; 95% CI: –3.27 to 31.13), and a significant drop from 2008 to 2020 (APC:–3.08%; 95% CI: –4.78 to–1.36) (Table S5, Supplemental Digital Content, https://links.lww.com/MD/R192; Fig. 5).

## 10. Urbanization-based differences

When stratified by urbanization level, AAMRs revealed a clear geographic gradient. The highest AAMR was reported in NonCore (Nonmetro) areas at 0.98 per 100,000 (95% CI: 0.91–1.06), accounting for 7.93% of total deaths. This was followed closely by Micropolitan (Nonmetro) regions with an AAMR of 0.87 (95% CI: 0.81–0.92) and 10.33% of deaths, and Small Metro areas at 0.76 (95% CI: 0.71–0.81) with 9.89% contribution. Medium Metro regions reported an AAMR of 0.73 (95% CI: 0.70–0.77) and made up 22.41% of deaths. Among more urbanized areas, Large Central Metro regions had an AAMR of 0.64 (95% CI: 0.61–0.66), representing the largest share (30.46%) of deaths, while Large Fringe Metro areas recorded the lowest AAMR at 0.55 (95% CI: 0.53–0.58) and 18.99% of total mortality.

Temporal trends varied significantly across these classifications. In Large Central Metro areas, a significant decline was observed from 1999 to 2004 (APC:–5.35%; 95% CI: –9.76 to–0.73), followed by a nonsignificant rise between 2004 and 2007 (APC: +18.98%; 95% CI: –2.24 to 44.79), and a strong and sustained significant decline from 2007 to 2020 (APC:–2.61%; 95% CI: –3.58 to–1.64). Large Fringe Metro areas experienced a significant increase in mortality from 1999 to 2006 (APC: +8.31%; 95% CI: 2.80–14.11), followed by a nonsignificant decline through 2020 (APC:–1.06%; 95% CI: –2.57 to 0.48). In Medium Metro areas, mortality significantly rose from 1999 to 2009 (APC: +4.24%; 95% CI: 1.23–7.35), followed by a decline from 2009 to 2020 (APC:–1.01%; 95% CI: –3.37 to 1.41), although not statistically significant.

**Figure 6.**
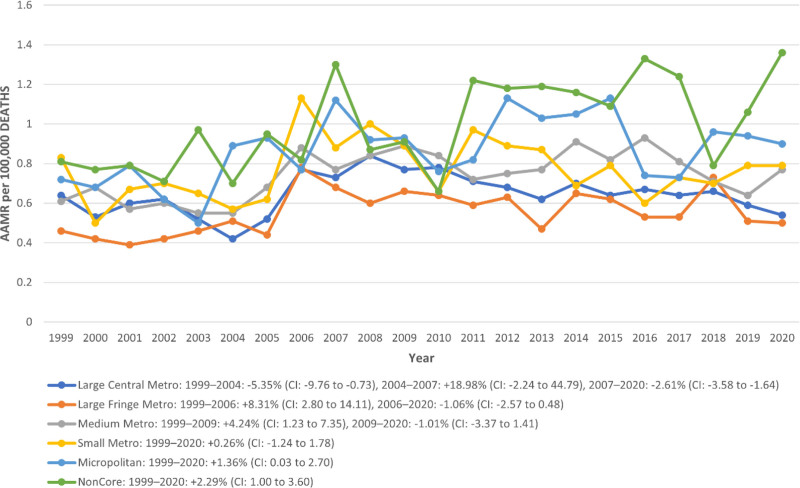
Trends in congenital malformation of urinary system AAMR stratified by urbanization. * indicates that the APC is significantly different from zero at the alpha = 0.05 level. Figure OA infographics. Central illustration: pediatric demographics and regional trends from CAKUT: A U.S. Population-Based Study from 1999 to 2020. AAMR = age-adjusted mortality rate, APC = annual percent change, CAKUT = congenital anomalies of the kidney and urinary tract.

A relatively stable nonsignificant trend was seen in Small Metro areas from 1999 to 2020 (APC: +0.26%; 95% CI: –1.24 to 1.78), while Micropolitan (Nonmetro) areas experienced a significant upward trend over the entire period (APC: +1.36%; 95% CI: 0.03–2.70). The sharpest increase was recorded in NonCore (Nonmetro) areas, with a significant annual rise of + 2.29% (95% CI: 1.00–3.60) from 1999 to 2020. These patterns suggest a growing disparity in mortality outcomes between rural and urban populations over time (Table S7, Supplemental Digital Content, https://links.lww.com/MD/R192; Fig. 6).

## 11. Subtype-specific mortality patterns

Sensitivity analysis revealed distinct mortality burdens across CAKUT categories: renal agenesis/dysgenesis (Q60) accounted for 52.5% of deaths (n = 4940), cystic kidney diseases (Q61) contributed 37.1% (n = 3490), and obstructive anomalies (Q62–Q64) represented 8.4% (n = 791). Within these categories, Potter syndrome (Q60.6) emerged as the single largest cause-specific mortality entity (17.0% of all deaths), while posterior urethral valves (PUV; Q64.2) demonstrated both clinical significance (2.7% of mortality) and striking male predominance (89% of cases).

## 12. Discussion

Our 22-year analysis reveals that mortality from congenital urinary system malformations among U.S. children remained relatively low overall but exhibited modest fluctuations over time. This pattern likely reflects advances in prenatal diagnosis and neonatal management. The widespread use of fetal ultrasound and enhanced newborn screening protocols have allowed for early identification of kidney and urinary tract anomalies, which in turn facilitates timely clinical interventions such as surgical planning and nephrologic surveillance.^[9–11]^ These developments have been especially beneficial for infants born with complex anomalies, leading to improved survival.^[12,13]^

Maternal health plays a crucial role in determining congenital anomaly outcomes. CAKUT have been consistently linked to maternal factors such as preexisting diabetes, obesity, poor glycemic control, and nutritional deficiencies.^[14–17]^ For instance, maternal obesity has been associated with an increased risk of severe urological anomalies, particularly PUV, which are known to cause obstructive uropathy and carry a high mortality risk if undiagnosed or untreated.^[18,19]^ Efforts aimed at improving maternal nutrition and glycemic control, especially in high-risk populations, could thus reduce the incidence and severity of urinary system malformations.^[15,20]^

National trends in maternal comorbidities may contribute to observed patterns. From 1999 to 2020, maternal obesity prevalence rose from 28% to 42%,^[21]^ while gestational diabetes increased by 56%.^[22]^ These conditions establish well-established risk factors for severe CAKUT phenotypes, particularly PUV (adjusted OR = 2.3; 95% CI: 1.7–3.1).^[23]^

Gender disparities in mortality from congenital urogenital anomalies were evident throughout our study period. Males exhibited significantly higher AAMR compared to females, which may be attributed to sex-specific anomalies such as PUV that occur exclusively in boys and are often more severe.^[24–26]^ These conditions can lead to life-threatening complications like renal failure and pulmonary hypoplasia. Despite these disparities, both sexes have benefited from medical advancements over time, as reflected in the overall declining trends in mortality, especially since 2007.^[27]^

Our findings also underscore significant racial and ethnic disparities in mortality. While NH White children represented most deaths due to population proportion, the AAMR for NH Black and Hispanic children was comparable or higher. These disparities may reflect complex interplays between genetics, socioeconomic factors, and healthcare access.^[28,29]^ For instance, variations in genes regulating nephrogenesis and inflammatory responses may predispose certain racial groups to more severe phenotypes of CAKUT. Furthermore, social determinants of health, such as lower household income, limited insurance coverage, and reduced access to prenatal care, disproportionately affect minority populations and exacerbate risks.^[30]^

Geographic and urbanization patterns further reveal disparities in care and outcomes. We observed that states with high poverty rates and limited healthcare infrastructure – such as Mississippi, South Dakota, and rural regions – had the highest AAMRs. Noncore and micropolitan counties demonstrated mortality rates nearly twice as high as those in large metro areas, suggesting that access to specialized pediatric urology and nephrology services is a significant determinant of survival. This urban–rural gap is consistent with prior literature emphasizing workforce shortages and delayed referrals in underserved areas.^[31,32]^

Public health responses must address these disparities through a multifaceted approach. Expanding prenatal ultrasound availability in underserved areas, enhancing maternal-child health (MCH) programs, and integrating obstetric care with pediatric nephrology/urology can significantly improve outcomes.^[33,34]^ MCH clinics that provide bundled services – ranging from preconception counseling to neonatal specialty referral – have shown promise in improving continuity of care.^[35]^ Furthermore, multivitamin supplementation and chronic disease management during pregnancy should be emphasized as cost-effective strategies to prevent CAKUT, particularly in low-income and minority communities.^[14,17]^

Addressing regional and socioeconomic disparities requires expanding pediatric subspecialist access through both physical deployment and telemedicine. Rural communities can benefit from hub-and-spoke models where regional centers provide remote consults to local clinicians, enabling early identification and management of urological anomalies. These services should be complemented by Medicaid expansion, transportation support, and targeted outreach to mitigate economic and logistical barriers to care.

Clinical management of CAKUT must follow standardized protocols that incorporate both surgical and pharmacologic interventions. For example, in children with vesicoureteral reflux, prophylactic antibiotics have been shown to reduce the incidence of urinary tract infections and subsequent renal scarring, as demonstrated in the RIVUR trial.^[12]^ In children with obstructive anomalies, early surgical intervention (e.g., valve ablation or pyeloplasty) is essential for preserving kidney function and preventing progression to end-stage renal disease. Moreover, pharmacologic therapies such as angiotensin-converting enzyme inhibitors have proven efficacy in reducing proteinuria and slowing renal decline in children with CAKUT-associated chronic kidney disease.^[36]^

To promote equity, culturally tailored public health campaigns are essential. These should focus on educating families about maternal risk factors (e.g., diabetes, teratogen exposure, poor nutrition) and the importance of early prenatal care. Partnerships with community health organizations and federal programs (e.g., WIC, Title V) can help disseminate these messages and improve maternal engagement. Importantly, ongoing surveillance through birth defects registries must be strengthened to monitor trends by race, region, and urbanicity and inform targeted policy decisions.^[37,38]^

In conclusion, while mortality due to congenital urogenital malformations remains low, significant disparities persist across sex, race/ethnicity, geography, and urbanization. The modest national decline in mortality since 2007 is encouraging and likely reflects enhanced prenatal detection, surgical innovations, and better neonatal care. However, continued investment in maternal health programs, pediatric subspecialist networks, health equity initiatives, and pharmacologic optimization are required. These interventions, aligned with public health goals and MCH frameworks, offer a pathway to further reduce mortality and improve outcomes for all children born with urinary malformations.

## 13. Limitations

This study has several limitations. Its retrospective design limits data quality control and may introduce biases inherent to secondary data analysis. Dependence on ICD codes poses risks of misclassification or underreporting, and changes in coding practices over time could affect trend consistency. While Q61 codes represent cystic developmental anomalies, their inclusion may overrepresent nonobstructive pathologies. This could potentially skew mortality burden estimates away from obstructive causes like PUV. However, our sensitivity analysis (excluding Q61 codes) demonstrated consistent temporal trends (AAPC + 0.62% vs + 0.59%), mitigating concerns about this classification bias. The absence of clinical details, including anomaly severity and treatment history, as well as unmeasured confounders like maternal health, prenatal care, and socioeconomic status, restricts analytic depth. Additionally, causal relationships cannot be established, and reporting variability and regional differences in pediatric healthcare services may limit the study’s generalizability.

## 14. Conclusion

In conclusion, the AAMRs from congenital urinary anomalies between 1999 and 2020 highlight significant advancements in prenatal diagnosis, neonatal care, maternal health, and healthcare systems. However, notable disparities persist, such as a 78% higher mortality rate in rural areas and an 89% male predominance in PUV cases. These disparities underscore the need for further research into the biological, sociocultural, genetic, and healthcare access factors contributing to these outcomes. To address the 78% higher mortality in rural regions, targeted interventions should include maternal diabetes screening programs, expanded telehealth access for prenatal diagnostics, and the development of a more robust pediatric nephrology workforce.

## Author contributions

**Conceptualization:** Muhammad Ibrahim, Malik Aqeel Ahmad, Fizza Inam, Fnu Sahil, Muhammad Khalid Afridi, Muhammad Husnain Ahmad, Muhammad Umair Ghafoor, Hanzala Zahid, Muhammad Bilal, Masab Ali.

**Data curation:** Malik Aqeel Ahmad, Fnu Sahil, Hanzala Zahid, Muhammad Bilal, Masab Ali.

**Methodology:** Muhammad Ibrahim, Muhammad Umair Ghafoor.

**Validation:** Fizza Inam, Raheel Ahmad, Fatima Arshad.

**Visualization:** Fizza Inam.

**Writing** – **original draft:** Muhammad Ibrahim, Fizza Inam, Fnu Sahil, Muhammad Khalid Afridi, Muhammad Husnain Ahmad, Fatima Arshad, Muhammad Umair Ghafoor, Hanzala Zahid, Muhammad Bilal, Masab Ali.

**Writing** – **review & editing:** Malik Aqeel Ahmad, Muhammad Khalid Afridi, Raheel Ahmad, Muhammad Husnain Ahmad, Fatima Arshad, Hanzala Zahid.

## Supplementary Material


