# Supplementary material for: Pediatric demographics and regional trends from congenital anomalies of the kidney and urinary tract: A U.S. population-based study from 1999 to 2020
Source: Medicine (Baltimore). 2026 Jan 23;105(4):e47314. doi: 10.1097/MD.0000000000047314 (PMC12851642; doi:10.1097/MD.0000000000047314)
Supplement: Supplementary file 1 [file medi-105-e47314-s001.pdf]

Supplementary Table 1: Frequency and age adjusted mortality rates per 100,000 deaths in pediatric population aged <15 in the United States, 1999 to 2020

**Category**	**Deaths**	**Population**	**Overall AAMR per 100,000 (95% CI)**
Total	9412	1336707436	0.68 (0.67–0.70)
**Sex**			
Male	6090	683360966	0.87 (0.85–0.89)
Female	3322	653346470	0.49 (0.47–0.51)
**Race**			
Black or African American	1752	222004493	0.76 (0.72–0.80)
White	7243	1018282374	0.70 (0.68–0.71)
Hispanic or Latino	2381	301252768	0.70 (0.67–0.73)
**Urbanization**			
Metropolitan	7694	1144506212	0.65 (0.64–0.66)
Non-Metropolitan	1718	192199378	0.9(0.86-0.94)
**Census Region**			
Census Region 1: Northeast	1070	223250176	0.48 (0.45–0.51)
Census Region 2: Midwest	2233	291560754	0.76 (0.73–0.79)
Census Region 3: South	4087	499636548	0.79 (0.76–0.81)
Census Region 4: West	2022	322259958	0.60 (0.57–0.62)

Supplementary Table 2: Annual age adjusted mortality rates per 100,000 deaths in pediatric population aged <15 in the United States, 1999 to 2020

Year	Age Adjusted Rate	Age Adjusted Rate Lower 95% Confidence Interval	Age Adjusted Rate Upper 95% Confidence Interval
1999	0.61	0.55	0.67
2000	0.55	0.49	0.61
2001	0.57	0.51	0.63
2002	0.59	0.53	0.65
2003	0.53	0.48	0.59
2004	0.55	0.49	0.61
2005	0.59	0.53	0.65
2006	0.82	0.75	0.89
2007	0.8	0.73	0.87
2008	0.79	0.72	0.86
2009	0.8	0.73	0.87
2010	0.73	0.67	0.8
2011	0.73	0.67	0.8
2012	0.75	0.68	0.82
2013	0.7	0.63	0.76
2014	0.77	0.7	0.84
2015	0.74	0.67	0.81
2016	0.73	0.66	0.79
2017	0.7	0.63	0.76
2018	0.72	0.65	0.79
2019	0.64	0.58	0.71
2020	0.68	0.61	0.74

Supplementary Table 3: Age adjusted mortality rates per 100,000 deaths stratified by sex in pediatric population aged < 15 in the United States, 1999 to 2020

Sex	Year	Age Adjusted Rate	Age Adjusted Rate Lower 95% Confidence Interval	Age Adjusted Rate Upper 95% Confidence Interval
Female	1999	0.48	0.4	0.56
Female	2000	0.41	0.34	0.49
Female	2001	0.38	0.31	0.45
Female	2002	0.47	0.39	0.55
Female	2003	0.31	0.25	0.38
Female	2004	0.41	0.34	0.48
Female	2005	0.41	0.34	0.48
Female	2006	0.64	0.55	0.73
Female	2007	0.55	0.47	0.64
Female	2008	0.59	0.5	0.67
Female	2009	0.62	0.54	0.71
Female	2010	0.47	0.39	0.55
Female	2011	0.5	0.42	0.58
Female	2012	0.55	0.46	0.63
Female	2013	0.51	0.43	0.59
Female	2014	0.6	0.51	0.69
Female	2015	0.56	0.48	0.64
Female	2016	0.47	0.39	0.55
Female	2017	0.48	0.4	0.55
Female	2018	0.55	0.46	0.63
Female	2019	0.48	0.4	0.56
Female	2020	0.46	0.38	0.54
Male	1999	0.75	0.65	0.84
Male	2000	0.7	0.6	0.79
Male	2001	0.77	0.67	0.87
Male	2002	0.71	0.62	0.81
Male	2003	0.75	0.66	0.85
Male	2004	0.65	0.56	0.74
Male	2005	0.8	0.7	0.9
Male	2006	0.99	0.88	1.1
Male	2007	1.02	0.91	1.13
Male	2008	1.01	0.9	1.12
Male	2009	0.99	0.88	1.1
Male	2010	0.99	0.88	1.1
Male	2011	0.98	0.87	1.09
Male	2012	0.97	0.86	1.08
Male	2013	0.9	0.8	1.01

Male	2014	0.94	0.83	1.05
Male	2015	0.91	0.8	1.01
Male	2016	0.97	0.86	1.08
Male	2017	0.9	0.8	1.01
Male	2018	0.89	0.78	0.99
Male	2019	0.8	0.7	0.91
Male	2020	0.88	0.77	0.98

Supplementary Table 4: Age adjusted mortality rates per 100,000 deaths stratified by race in pediatric population aged < 15 in the United States, 1999 to 2020

Race	Year	Age Adjusted Rate	Age Adjusted Rate Lower 95% Confidence Interval	Age Adjusted Rate Upper 95% Confidence Interval
Black or African American	1999	0.68	0.53	0.87
Black or African American	2000	0.52	0.39	0.69
Black or African American	2001	0.63	0.48	0.81
Black or African American	2002	0.44	0.32	0.59
Black or African American	2003	0.59	0.45	0.77
Black or African American	2004	0.53	0.4	0.7
Black or African American	2005	0.7	0.55	0.89
Black or African American	2006	1.05	0.85	1.25
Black or African American	2007	1	0.81	1.19
Black or African American	2008	0.85	0.68	1.04
Black or African American	2009	0.91	0.74	1.11
Black or African American	2010	0.84	0.67	1.03
Black or African American	2011	0.87	0.7	1.06

Black or African American	2012	0.8	0.64	0.99
Black or African American	2013	0.71	0.56	0.9
Black or African American	2014	0.82	0.66	1.02
Black or African American	2015	0.8	0.64	0.99
Black or African American	2016	0.98	0.8	1.17
Black or African American	2017	0.72	0.57	0.9
Black or African American	2018	0.93	0.75	1.13
Black or African American	2019	0.72	0.56	0.91
Black or African American	2020	0.66	0.52	0.84
White	1999	0.59	0.52	0.66
White	2000	0.58	0.51	0.65
White	2001	0.6	0.53	0.67
White	2002	0.62	0.55	0.7
White	2003	0.53	0.47	0.6
White	2004	0.56	0.49	0.63
White	2005	0.59	0.52	0.66
White	2006	0.8	0.72	0.88
White	2007	0.8	0.73	0.88
White	2008	0.84	0.75	0.92
White	2009	0.82	0.74	0.91
White	2010	0.75	0.67	0.83
White	2011	0.75	0.67	0.83
White	2012	0.75	0.67	0.83
White	2013	0.74	0.66	0.82
White	2014	0.8	0.72	0.89
White	2015	0.76	0.68	0.84
White	2016	0.7	0.63	0.78

White	2017	0.71	0.63	0.79
White	2018	0.71	0.63	0.79
White	2019	0.66	0.58	0.73
White	2020	0.71	0.63	0.79
Hispanic or Latino	1999	0.68	0.54	0.84
Hispanic or Latino	2000	0.66	0.52	0.82
Hispanic or Latino	2001	0.62	0.49	0.76
Hispanic or Latino	2002	0.64	0.51	0.79
Hispanic or Latino	2003	0.53	0.42	0.67
Hispanic or Latino	2004	0.57	0.46	0.71
Hispanic or Latino	2005	0.67	0.54	0.8
Hispanic or Latino	2006	0.8	0.66	0.95
Hispanic or Latino	2007	0.79	0.66	0.93
Hispanic or Latino	2008	0.93	0.78	1.07
Hispanic or Latino	2009	0.84	0.7	0.98
Hispanic or Latino	2010	0.79	0.65	0.92
Hispanic or Latino	2011	0.71	0.58	0.84
Hispanic or Latino	2012	0.7	0.57	0.83
Hispanic or Latino	2013	0.61	0.5	0.75
Hispanic or Latino	2014	0.75	0.62	0.89
Hispanic or Latino	2015	0.75	0.61	0.88
Hispanic or Latino	2016	0.7	0.57	0.82
Hispanic or Latino	2017	0.69	0.56	0.82
Hispanic or Latino	2018	0.7	0.56	0.83
Hispanic or Latino	2019	0.66	0.53	0.79

Hispanic or Latino	2020	0.69	0.56	0.82

Supplementary Table 5: Age adjusted mortality rates per 100,000 deaths stratified by census in pediatric population aged < 15 in the United States, 1999 to 2020

Census Region	Year	Age Adjusted Rate	Age Adjusted Rate Lower 95% Confidence Interval	Age Adjusted Rate Upper 95% Confidence Interval
Census Region 1: Northeast	1999	0.44	0.32	0.59
Census Region 1: Northeast	2000	0.46	0.34	0.61
Census Region 1: Northeast	2001	0.48	0.36	0.63
Census Region 1: Northeast	2002	0.39	0.28	0.53
Census Region 1: Northeast	2003	0.3	0.2	0.42
Census Region 1: Northeast	2004	0.3	0.2	0.42
Census Region 1: Northeast	2005	0.41	0.29	0.55
Census Region 1: Northeast	2006	0.62	0.47	0.79
Census Region 1: Northeast	2007	0.57	0.44	0.74
Census Region 1: Northeast	2008	0.54	0.41	0.7
Census Region 1: Northeast	2009	0.61	0.47	0.79
Census Region 1: Northeast	2010	0.6	0.46	0.78
Census Region 1: Northeast	2011	0.6	0.46	0.77
Census Region 1: Northeast	2012	0.5	0.37	0.66
Census Region 1: Northeast	2013	0.37	0.26	0.51
Census Region 1: Northeast	2014	0.51	0.38	0.68
Census Region 1: Northeast	2015	0.41	0.3	0.56
Census Region 1: Northeast	2016	0.5	0.37	0.66
Census Region 1: Northeast	2017	0.48	0.35	0.64
Census Region 1: Northeast	2018	0.68	0.53	0.87

Census Region 1: Northeast	2019	0.41	0.29	0.56
Census Region 1: Northeast	2020	0.41	0.29	0.57
Census Region 2: Midwest	1999	0.65	0.52	0.8
Census Region 2: Midwest	2000	0.56	0.44	0.7
Census Region 2: Midwest	2001	0.64	0.51	0.79
Census Region 2: Midwest	2002	0.7	0.56	0.85
Census Region 2: Midwest	2003	0.66	0.53	0.82
Census Region 2: Midwest	2004	0.48	0.37	0.61
Census Region 2: Midwest	2005	0.7	0.56	0.85
Census Region 2: Midwest	2006	0.79	0.64	0.94
Census Region 2: Midwest	2007	0.98	0.81	1.14
Census Region 2: Midwest	2008	0.98	0.82	1.15
Census Region 2: Midwest	2009	0.9	0.74	1.06
Census Region 2: Midwest	2010	0.66	0.53	0.82
Census Region 2: Midwest	2011	0.78	0.63	0.93
Census Region 2: Midwest	2012	0.94	0.77	1.11
Census Region 2: Midwest	2013	0.75	0.6	0.91
Census Region 2: Midwest	2014	0.97	0.8	1.14
Census Region 2: Midwest	2015	1.03	0.85	1.2
Census Region 2: Midwest	2016	0.73	0.59	0.9
Census Region 2: Midwest	2017	0.78	0.63	0.93
Census Region 2: Midwest	2018	0.78	0.63	0.95
Census Region 2: Midwest	2019	0.76	0.61	0.93

Census Region 2: Midwest	202 0	0.73	0.59	0.9
Census Region 3: South	199 9	0.65	0.54	0.76
Census Region 3: South	200 0	0.63	0.52	0.74
Census Region 3: South	200 1	0.62	0.51	0.72
Census Region 3: South	200 2	0.59	0.49	0.69
Census Region 3: South	200 3	0.66	0.55	0.76
Census Region 3: South	200 4	0.72	0.61	0.83
Census Region 3: South	200 5	0.7	0.59	0.81
Census Region 3: South	200 6	1.06	0.92	1.19
Census Region 3: South	200 7	0.86	0.75	0.98
Census Region 3: South	200 8	0.86	0.74	0.97
Census Region 3: South	200 9	0.9	0.78	1.02
Census Region 3: South	201 0	0.83	0.71	0.95
Census Region 3: South	201 1	0.82	0.7	0.93
Census Region 3: South	201 2	0.82	0.71	0.94
Census Region 3: South	201 3	0.91	0.79	1.04
Census Region 3: South	201 4	0.88	0.76	1
Census Region 3: South	201 5	0.79	0.67	0.9
Census Region 3: South	201 6	0.88	0.76	1
Census Region 3: South	201 7	0.8	0.69	0.92
Census Region 3: South	201 8	0.83	0.71	0.95
Census Region 3: South	201 9	0.8	0.68	0.91
Census Region 3: South	202 0	0.82	0.71	0.94

Census Region 4: West	1999	0.71	0.57	0.85
Census Region 4: West	2000	0.5	0.39	0.63
Census Region 4: West	2001	0.54	0.43	0.67
Census Region 4: West	2002	0.62	0.5	0.76
Census Region 4: West	2003	0.45	0.35	0.57
Census Region 4: West	2004	0.47	0.36	0.6
Census Region 4: West	2005	0.53	0.42	0.66
Census Region 4: West	2006	0.65	0.52	0.78
Census Region 4: West	2007	0.68	0.55	0.8
Census Region 4: West	2008	0.73	0.6	0.87
Census Region 4: West	2009	0.7	0.56	0.83
Census Region 4: West	2010	0.74	0.6	0.88
Census Region 4: West	2011	0.7	0.56	0.83
Census Region 4: West	2012	0.72	0.59	0.86
Census Region 4: West	2013	0.58	0.46	0.72
Census Region 4: West	2014	0.64	0.52	0.79
Census Region 4: West	2015	0.63	0.51	0.77
Census Region 4: West	2016	0.65	0.52	0.78
Census Region 4: West	2017	0.59	0.47	0.72
Census Region 4: West	2018	0.51	0.4	0.65
Census Region 4: West	2019	0.48	0.37	0.61
Census Region 4: West	2020	0.55	0.43	0.68

Supplementary Table 6: State wise age adjusted mortality rates per 100,000 deaths in pediatric population aged < 15 in the United States, 1999 to 2020

State	Age Adjusted Rate	Age Adjusted Rate Lower 95% Confidence Interval	Age Adjusted Rate Upper 95% Confidence Interval	% of Total Deaths
Alabama	1.02	0.88	1.16	2.22%
Alaska	0.75	0.5	1.1	0.29%
Arizona	0.73	0.63	0.82	2.29%
Arkansas	1.02	0.85	1.2	1.42%
California	0.56	0.52	0.6	10.35%
Colorado	0.57	0.47	0.67	1.40%
Connecticut	0.44	0.34	0.57	0.64%
Delaware	0.53	0.33	0.83	0.21%
District of Columbia	1.35	0.95	1.85	0.39%
Florida	0.73	0.67	0.8	5.81%
Georgia	0.69	0.61	0.77	3.35%
Hawaii	0.55	0.38	0.78	0.35%
Idaho	0.63	0.47	0.84	0.52%
Illinois	0.61	0.55	0.68	3.70%
Indiana	0.91	0.8	1.02	2.85%
Iowa	0.77	0.62	0.92	1.08%
Kansas	0.93	0.77	1.1	1.32%
Kentucky	0.88	0.75	1.02	1.80%
Louisiana	0.95	0.82	1.08	2.16%
Maine	0.49	0.31	0.74	0.23%
Maryland	0.53	0.44	0.62	1.42%
Massachusetts	0.33	0.26	0.41	0.90%
Michigan	0.75	0.67	0.84	3.30%
Minnesota	0.66	0.56	0.77	1.68%
Mississippi	1.19	1.01	1.37	1.75%
Missouri	0.86	0.75	0.98	2.38%
Montana	0.67	0.44	0.97	0.29%
Nebraska	0.84	0.66	1.05	0.78%
Nevada	0.69	0.55	0.86	0.88%
New Hampshire	Unreliable	0.23	0.62	0.19%

New Jersey	0.41	0.35	0.48	1.63%
New Mexico	0.71	0.55	0.9	0.70%
New York	0.45	0.4	0.5	4.06%
North Carolina	0.75	0.66	0.83	3.30%
North Dakota	0.7	0.44	1.06	0.23%
Ohio	0.83	0.75	0.91	4.24%
Oklahoma	0.93	0.78	1.07	1.74%
Oregon	0.59	0.47	0.72	0.98%
Pennsylvania	0.64	0.57	0.71	3.37%
Rhode Island	0.59	0.37	0.88	0.24%
South Carolina	0.76	0.64	0.88	1.60%
South Dakota	0.97	0.68	1.33	0.40%
Tennessee	0.98	0.87	1.1	2.85%
Texas	0.75	0.71	0.8	10.55%
Utah	0.82	0.69	0.96	1.49%
Vermont	Unreliable	0.23	0.89	0.11%
Virginia	0.6	0.52	0.69	2.20%
Washington	0.57	0.48	0.65	1.80%
West Virginia	0.88	0.67	1.14	0.64%
Wisconsin	0.7	0.59	0.8	1.76%
Wyoming	Unreliable	0.32	0.98	0.15%

Supplementary Table 7: Age adjusted mortality rates per 100,000 deaths stratified by urbanization in pediatric population aged < 15 in the United States, 1999 to 2020

urbanization	Year	Age Adjusted Rate	Age Adjusted Rate Lower 95% Confidence Interval	Age Adjusted Rate Upper 95% Confidence Interval
Metropolitan	1999	0.59	0.53	0.66
Metropolitan	2000	0.53	0.46	0.59
Metropolitan	2001	0.53	0.47	0.59
Metropolitan	2002	0.58	0.51	0.65
Metropolitan	2003	0.51	0.45	0.57
Metropolitan	2004	0.51	0.45	0.57
Metropolitan	2005	0.54	0.48	0.6
Metropolitan	2006	0.82	0.75	0.9
Metropolitan	2007	0.73	0.66	0.8
Metropolitan	2008	0.79	0.71	0.86
Metropolitan	2009	0.78	0.7	0.85
Metropolitan	2010	0.74	0.67	0.81
Metropolitan	2011	0.7	0.62	0.77
Metropolitan	2012	0.71	0.64	0.79
Metropolitan	2013	0.64	0.57	0.71
Metropolitan	2014	0.73	0.65	0.8
Metropolitan	2015	0.69	0.62	0.76
Metropolitan	2016	0.69	0.62	0.76
Metropolitan	2017	0.66	0.59	0.73

Metropolitan	2018	0.7	0.62	0.77
Metropolitan	2019	0.6	0.53	0.67
Metropolitan	2020	0.61	0.54	0.68
Non-Metropolitan	1999	0.75	0.58	0.96
Non-Metropolitan	2000	0.72	0.55	0.92
Non-Metropolitan	2001	0.77	0.6	0.98
Non-Metropolitan	2002	0.66	0.5	0.86
Non-Metropolitan	2003	0.69	0.53	0.89
Non-Metropolitan	2004	0.77	0.6	0.98
Non-Metropolitan	2005	0.93	0.74	1.15
Non-Metropolitan	2006	0.79	0.62	1
Non-Metropolitan	2007	1.19	0.97	1.41
Non-Metropolitan	2008	0.86	0.68	1.07
Non-Metropolitan	2009	0.93	0.74	1.15
Non-Metropolitan	2010	0.7	0.54	0.9

Non-Metropolitan	2011	0.97	0.77	1.2
Non-Metropolitan	2012	1.14	0.92	1.39
Non-Metropolitan	2013	1.07	0.86	1.32
Non-Metropolitan	2014	1.07	0.86	1.32
Non-Metropolitan	2015	1.11	0.89	1.36
Non-Metropolitan	2016	0.97	0.77	1.21
Non-Metropolitan	2017	0.92	0.72	1.15
Non-Metropolitan	2018	0.88	0.69	1.11
Non-Metropolitan	2019	0.98	0.78	1.23
Non-Metropolitan	2020	1.08	0.86	1.34
